# Current practice of hospital-based palliative care teams: Advance care planning in advanced stages of disease: A retrospective observational study

**DOI:** 10.1371/journal.pone.0288514

**Published:** 2024-02-29

**Authors:** Iris van Doorne, Dick L. Willems, Nadine Baks, Jelle de Kuijper, Bianca M. Buurman, Marjon van Rijn

**Affiliations:** 1 Amsterdam UMC Location University of Amsterdam, Internal Medicine, Section of Geriatric Medicine, Amsterdam, The Netherlands; 2 Amsterdam Public Health, Aging and Later Life, Amsterdam, The Netherlands; 3 Center of Expertise Urban Vitality, Faculty of Health, Amsterdam University of Applied Science, Amsterdam, the Netherlands; 4 Amsterdam UMC Location University of Amsterdam, General Practice, Section of Medical Ethics, Amsterdam, The Netherlands; 5 Amsterdam UMC Location Vrije Universiteit, Medicine for Older People, Amsterdam, The Netherlands; University of Central Florida, UNITED STATES

## Abstract

**Background:**

Specialist palliative care teams are consulted during hospital admission for advice on complex palliative care. These consultations need to be timely to prevent symptom burden and maintain quality of life. Insight into specialist palliative care teams may help improve the outcomes of palliative care.

**Methods:**

In this retrospective observational study, we analyzed qualitative and quantitative data of palliative care consultations in a six-month period (2017 or 2018) in four general hospitals in the northwestern part of the Netherlands. Data were obtained from electronic medical records.

**Results:**

We extracted data from 336 consultations. The most common diagnoses were cancer (54.8%) and organ failure (26.8%). The estimated life expectancy was less than three months for 52.3% of all patients. Within two weeks after consultation, 53.2% of the patients died, and the median time until death was 11 days (range 191) after consultation. Most patients died in hospital (49.4%) but only 7.5% preferred to die in hospital. Consultations were mostly requested for advance care planning (31.6%). End-of-life preferences focused on last wishes and maintaining quality of life.

**Conclusion:**

This study provides detailed insight into consultations of palliative care teams and shows that even though most palliative care consultations were requested for advance care planning, consultations focus on end-of-life care and are more crisis-oriented than prevention-oriented. Death often occurs too quickly after consultation for end-of-life preferences to be met and these preferences tend to focus on dying. Educating healthcare professionals on when to initiate advance care planning would promote a more prevention-oriented approach. Defining factors that indicate the need for timely palliative care team consultation and advance care planning could help timely identification and consultation.

## Introduction

Because the population is aging, more older people with chronic diseases are being admitted each year. Approximately 65% of older people undergo care transitions in the last three months of life, most frequently to hospital [2]. Whilst most people (60%) prefer to die at home, for 30% of these people with chronic diseases, this wish is not fulfilled as these people die in hospital or in a nursing home [1].

Palliative care can benefit people with life threatening illnesses. It can improve the quality of life for both the patient and their family by treating physical, phychosocial, and spiritual problems [2]. If palliative care is initiated during early stages of disease, it can prevent suffering by guiding the patient’s decisions on their end-of-life preferences. These preferences can be discussed during advance care planning (ACP), which is defined as ‘the ability to enable individuals to define goals and preferences for future medical treatment and care, to discuss these goals and preferences with family and healthcare providers, and to record and review these preferences if appropriate’ [3]. ACP is considered an essential part of good palliative care [4] and if it is not performed during early stage of disease, treatment decisions are often crisis-oriented and do not meet end-of-life preferences [5].

One crisis-oriented decision is acute hospital admission, which many older people experience in the last phase of life [6]. After being admitted to hospital in the Netherlands, these older patients are treated by healthcare professionals who often do not have specialist expertise in palliative care, such as junior doctors, medical specialists and nurses, according to the generalist plus specialist model [7]. In this model, general healthcare professionals are primarily responsible for providing palliative care. However, palliative care can be complex and ACP conversations can be difficult, and is only covered limited in the curriculum for healthcare professionals [8].

To help in these complex palliative care situations, a specialist palliative care team can be consulted. Since 2017, every Dutch hospital providing oncology care must have a palliative care team [7]. These palliative care teams can be consulted for advice on treating inpatients and outpatients at different stages of disease and life, and these consultations have been shown to promote quality of care and satisfaction with care [9–11].

Most studies on palliative care teams have focused on patients with cancer; therefore, detailed insight into current practice of palliative care teams in the general population, including patients with organ failure, is less known. Furthermore, to our knowledge, the end-of-life preferences discussed during these consultation are currently unknown. Greater insight into the use, content, and timing of palliative care team consultations could help improve palliative care. In this restrospective observational study, we assessed all palliative care team consultations performed in a six-month time period. Our aims were to determine which patients received a palliative care team as well as the timing, content, and follow-up outcomes of the consultation and to explore the wishes and end-of-life preferences of patients receiving a palliative care team consultation.

## Methods

We performed a retrospective observational descriptive study in which we analyzed both quantitative and qualitative data. This study was conducted in four general hospitals in the northwestern part of the Netherlands. In each hospital, we collected data from all consultations performed within a six months period (January—June) in 2017 or 2018. All first-time consultations for patients ≥18 years old were included. There were no exclusion criteria. The results are reported according to the Strengthening the Reporting of Observational studies in Epidemiology (STROBE) guidelines [12].

### Ethical considerations

The study protocol was assessed by the medical ethical board of the OLVG hospital Amsterdam and was exempt from ethical approval as it was found to not have to comply with the Medical research Involving Human Subject Act since the study only included observational patient data, informed consent was not required for this retrospective data collection since all data were analyzed and stored anonymously and was waived by the medical ethical board of the OLVG hospital Amsterdam (ACWO-MEC 16u.653/WCHJ/ WO 16.533). All methods were carried out in accordance with the declaration of Helsinki. Data processing was performed according to the General Data Protection (Algemene Verordening Gegevensbescherming (AVG) in Dutch.

### Data collection

Every participating hospital provided a list of patients who received a palliative care team consultation during the six-month study period. Data were collected from the electronic medical records. Since one participating hospital shifted from handwritten patient records to electronic medical record in 2017, in this hospital data on consultations in 2018 were collected.

#### Patient characteristics

We extracted patient characteristics from the electronic medical record, including demographic characteristics, main life threatening illness, prescribed medication, estimated life expectancy, WHO/ECOG performance status [13], and healthcare use in the six months before consultation. Healthcare use included emergency room (ER) visits and unplanned hospital admission.

#### Mortality and place of death

We collected data on the patients’ preferred place of death and the eventual place and date of deaths from the hospital electronic medical record.

#### Consultation characteristics

Data on consultation content included the referring specialist, reason for and type of consultation, timing of consultation, and the number of visits and follow-up contacts by palliative care team members.

#### End-of-life preferences and wishes before death

We collected data on end-of-life preferences and wishes before death from reports written by the palliative care team consultant in the electronic medical record. Quotes were taken literatim from the reports.

### Analysis

#### Quantitative data

Standard descriptive statistics were used to describe patient and consultation characteristics. Data were analyzed using IBM SPSS Statistics (Version 26). Quantitative data included all patient and consultation characteristics except for the end-of-life preferences and wishes.

#### Qualitative data

End-of-life preferences and wishes before death reported by the palliative care team consultant were coded and categories were identified [14]. We used MaxQDA 2020 for this analysis.

## Results

### Patient characteristics

We collected data from 336 new palliative care team consultations, meaning that these patients were not introduced to the palliative care team at an earlier stage. The number of consultations per hospital over the six-month period varied: 27, 62, 64, and 183. Patients had a mean age of 75.1 years (SD 11.6) and 54.8% were male. The most common main diagnosis was cancer (54.8%), followed by organ failure (26.8%) and surgical problems (10.7%). In the six months prior to the palliative care team consultation, 47.9% of patients had visited the ER and 46.1% had been acutely admitted to hospital. Most patients (40.2%) were restricted to their bed or chair at the time of consultation, and 52.3% had an estimated life expectancy of less than three months (Table 1).

**Table 1:** Patient characteristics.

Patient characteristics	N = 336
Age (years), mean (SD)	75.1 (11.6)
Male, N (%)	184 (54.8)
Main diagnosis, N (%)	
Cancer	184 (54.8)
Organ failure	90 (26.8)
Surgical problems and complications	36 (10.7)
Frailty and neurological problems	26 (7.7)
ER visit in the six months prior to consultation, N (%)	161 (47.9)
Acute hospital admission in the six months prior to consultation, N (%)	155 (46.1)
Use of opioids, N (%)	175 (52.1)
Prognosis (estimated life expectancy), N (%)	(N = 306)
Days	56 (18.3)
Weeks	21 (6.9)
< 3 months	83 (27.1)
< 6 months	9 (2.9)
< 1 year	54 (17.6)
> 1 year	1 (0.3)
Unknown	82 (26.8)
WHO/ECOG performance status, N (%)	(N = 271)
0	8 (3.0)
1	22 (8.2)
2	41 (15.1)
3:	91 (33.8)
4	109 (40.2)

### Mortality

Within six months after consultation, 69.9% of the patients had died. The median time until death was 11 days (range 191) after consultation. Most patients (86%) died within three months after consultation, 37.4% within one week and 53.2% within two weeks after consultation. Most patients died in hospital (49.4%) during the admission at which the palliative care team was consulted, whilst only 7.5% of patients said they wanted to die in hospital. Most patients (62.8%) preferred to die at home but only 20.6% died at home (Table 2).

**Table 2:** Mortality and place of death.

Mortality and place of death	N = 336
Deceased during hospital admission at time of consultation, N (%)	84 (25)
Deceased within six months after consultation, N (%)	235 (70)
Within one week after consultation	88 (37.4)
Within two weeks after consultation	125 (53.2)
Within three months after consultation	202 (86.0)
Time until death after consultation (days), Median [range]	11 [3–36]
Preferred place of death, N (%)	(N = 172)
Home	116 (62.4)
Hospital	14 (7.5)
Hospice	35 (18.8)
Care home	7 (3.8)
Place of death, N (%)	(N = 170)
Home	35 (20.6)
Hospital	84 (49.4)
Hospice	45 (26.5)
Care home	6 (3.5)

### Consultation characteristics and follow-up

Most requests for consultation came from oncologists/hematologists (24.7%) and surgeons (19.9%) and most were for inpatients (83.6%). The main reasons for consultation were ACP (31.5%) and to get advice on patients’ palliative care needs (31%) or care needs in the dying phase (12.2%). Most consultations (63.1%) comprised a one-time visit of the nurse specialist and 61.9% included a discussion about the patient at a multidisciplinary team meeting. The supervisor of the palliative care team visited the patient in person in 27.4% of consultations. The preferred place of death was discussed with 55.9% of all patients. After the initial consultation visit, 55.9% of patients had follow-up contact with a member of the palliative care team, most often in the hospital (73.8%) (Table 3).

**Table 3:** Consultation characteristics and follow-up.

Consultation characteristics	N = 336
Consultation requester, N (%)	
Oncologist/hematologist	83 (24.7)
Surgeon	67 (19.9)
Pulmonologist	63 (18.8)
Cardiologist	33 (9.8)
Specialist for internal diseases/geriatrician	42 (12.5)
Nurse (specialist)/spiritual counselor	10 (3.0)
Patient/informal caregiver	2 (0.6)
Other	36 (10.7)
Reason for consultation as described by requester, N (%)	
Advance care planning	106 (31.5)
Advice on symptom management	49 (14.6)
Guidance/advice during palliative phase	104 (31)
Guidance/advice during dying phase	41 (12.2)
Medication advice	12 (3.6)
Advice concerning care transitions	11 (3.3)
Other	13 (3.9)
Type of consultation, N (%)	
Full consult (patient visited by nurse specialist and supervisor and discussed during MDT)	70 (20.8)
Full consult without visit from supervisor	208 (61.9)
Consult without MDT	27 (8.0)
Telephone advice	8 (1.2)
Other	23 (6.9)
Inpatient consultation, N (%)	281 (83.6)
Number of visits by nurse specialist during consultation, N (%)	
0	28 (8.4)
1	212 (63.1)
≥ 2	95 (28.3)
Number of visits by supervisor during consultation, N (%)	
0	240 (71.4)
1	89 (26.5)
2	3 (0.9)
Preferred place of death discussed during consultation, N (%)	186 (55.9)
	N = 252^a^
Follow-up contact between patient and palliative care team, N (%)	141 (55.9)
In-person contact	104 (73.8)
Telephone contact	52 (36.9)

^*a*^ These 252 patients did not die during the same hospital admission as the consultation

MDT = multidisciplinary team meeting

#### End-of-life preferences and wishes before death

End-of-life preferences and wishes before death were discussed and reported in 145 (43%) consultations. In 22.1% of consultations, patients noted they had no preferences or wishes before death, half of these cases were because the patient did not want to talk or think about the end of their life.

We extracted several categories from the palliative care team reports about end-of-life preferences and wishes before death.

#### Future treatment, organization, and transition of care

For one patient, preventing more hospital admissions was discussed. The most common wish regarding transition of care was to go home. Some patients did not explain this wish while others said they wanted to go home to be with family or to die at home. Some patients said they wanted to go to a hospice. Patients also wanted clarity about their treatment options, the opportunity to discontinue treatment if they wanted to, and clear statements about why they wanted to stop treatment.

“*Wants to undergo treatment*, *mild side effects are not a problem for him but in case of serious side effects and especially severe pain*, *he wants euthanasia*.*”*

#### Quality of life

For quality of life, patients wanted to maintain control, enjoy life, and not suffer from symptoms, particularly pain and dyspnea.

“*The most important thing is to be as comfortable as possible*, *and to experience as little symptom burden as possible”*

#### Last wishes

Last wishes included activities, seeing and saying goodbye to family, and euthanasia. Reported activities ranged from specific one-time activities to more general long-term activities. These long-term activities mainly included holidays and being able to perform activities in general. In many cases, the patient or palliative care team consultant questioned the feasibility of these wishes.

*“He says he still wants to do many things. He has many chores in and around the house waiting to be done. He mentioned so many chores, I indicated I thought that was not realistic and that it’s best to focus on doing the things that bring joy and peace.”*

Enjoying life was a general, non-concrete wish reported by patients. Keeping control of life and dying was also an important topic.

Patients mentioned family, friends, and pets in their last wishes. For many, seeing and being with a loved one before death, sometimes to say goodbye, was important. Others mentioned important days and events they wanted to attend, such as anniversaries and birthdays.

*“The biggest wish is to be able to see grandchildren grow up and experience the upcoming birth of a grandchild.”*

Lastly, many patients mentioned euthanasia in their last wishes; for some this was a short-term wish and for others it was something they wanted in the future.

“*Wants to say goodbye to his children before euthanasia starts*.*”*

## Discussion

In this retrospective obervational study, we analyzed all palliative care team consultations performed in four hospitals in the Netherlands within a six-month period. Our findings suggest that palliative care consultations usually take place during the end-of-life phase, since most patients died within three months after their consultation. A quarter of patients died during the hospital admission at which the palliative care team was consulted, and half died within one or two weeks after consultation. This meant that ACP, which was often the reason for consultation, could not be arranged in time. This could have contributed to many patients not dying in their prefered place of death. In addition, many reported end-of-life wishes focused on the final days and weeks before death and rarely had to do with treatment. The feasibility of these wishes was sometimes questioned.

### Interpretation of findings

Many studies have focused on palliative care in oncology patients [15–18]. In our sample, only half of the patients were diagnosed with cancer. As in previous research [9], we found that most consultations were requested for patients in the last three months of life. This suggests that palliative care continues to focus on late stage end-of-life care, as previously reported by Dalgaard et al. [19]. Patients who received a palliative care team consultation were often acutely hospitalized and severely restricted in daily activities, suggesting a more ad-hoc approach to palliative care that does not recognize the need for palliative care timely enough [3].

Although most patients in our study were diagnosed with a life threatening illness and had been acutely admitted to hospital in the six months before the consultation, identifying the need for palliative care still appears to be difficult. Previously reported barriers to identifying the need for palliative care include an uncertain prognosis, lack of knowledge about palliative care, and lack of consensus between healthcare professionals [19–21].

Although palliative care was initiated during advanced stages of disease, ACP was the most common reason for palliative care team consultation in our study. This high proportion of consultations for ACP suggest that general physicians have difficulty discussing end-of-life matters and are reluctant to start these conversations themselves, eventhough general physicians should primarly should undertake these discussions themselves. In addition, the increasing attention placed on ACP [22] could explain this high proportion of consultations. ACP is a complex process, for which often multiple conversations are needed for patients to fully explore their end-of-life preferences and make end-of-life treatment decisions [3]. It is questionable if the ACP conversations reported here were sufficient to serve this purpose.

One of the goals of ACP is to reduce overtreatment [23]. For many patients in our study, the hospital admission at which the palliative care team was consulted was their last care transition before death. If the palliative care team had been consulted during an earlier hospital admission (half of the patients had been admitted to hospital in the previous six months), then perhaps proactive communication could have avoided this last admission for some patients [24]. This would possibly also reduce the number of in-hospital deaths, which is often a goal of ACP [25]. In our study, 62% of patients wanted to die at home, which is in line with previous studies [1, 26, 27]. However, in our study, most patients actually died in the hospital, although very few patients wanted this. The congruence between the preferred place of death and the actual place of death was lower in our study than reported in previous studies [1]. This could be related to the more advanced stages of disease and the limited time until death in our patients, which cannot be compared to patients who did not receive a palliative care team consultation.

The end-of-life preferences reported by the patients in our study reflected the severity and advanced stages of their disease. In all themes, end-of-life wishes included things the patient wanted to do before death and were often one-time and final wishes. Reconsidering these wishes was not possible in most cases.

The underlying goals of ACP were described by Fleuren et al. [23] in a systematic review. These goals include improving quality of care, preparing for the end-of-life, and respecting patients’ autonomy. These goals could be achieved by preparing patients for ‘in-the-moment decisions’. Patients in our study were already ‘in-the-moment’, and were not able to make treatment decisions anymore. As a result, the themes ‘future treatment’ and ‘wishes regarding dying and death’ were focused on preparation for the end-of-life limitedly and often did not include treatment decision making. The theme ‘quality of life’ is comparable to previously described end-of-life wishes of patients in the dying phase [28], in which symptom management was important.

### Strenghts and limitations

A strength of our study is that we included all palliative care team consultations rather than focusing on a specific patient population. This is in contrast to previous studies that often focused on patients with cancer or other specific diseases. Therefore, we provide insight into palliative care team consulation in a broader more general population. Furthermore, including four hospitals gave a sample that is representative of the northwestern part of the Netherlands, reflecting urban as well as rural parts. The detailed data collection provided useful information on the characteristics of patients who receive palliative care team consultations and a deeper insight into the content of palliative care team consultations, including the end-of-life preferences and wishes of patients.

However, our study also has limitations. First, all data were obtained from the patients’ electronic medical record and focused on reports written by the palliative care team consultant, so the completeness of our qualitative data depended on completeness of the healthcare providers’ report. Some topics may have been discussed with the patient before the consultation or may not have been included in the electronic medical record. In addition, the patients’ preferences we collected were described by members of the palliative care team and may have been misinterpreted. Second, we could not track down the place of death for all patients, particularly those who did not die in the hospital, however, we do know the place of death for 72% of all patients, which we consider to be sufficient.

Third, the number of consultations varied widely between the included hospitals so our findings might be mostly contributable to hospitals that conducted the most consultations. To improve the generalizability of our findings, this study should be performed in all parts of the Netherlands or in more countries.

In conclusion, our study shows that timely identification of palliatiave care needs remain to be lacking. Palliative care consultations are still focused on end-of-life care and are more crisis-oriented than prevention-oriented. Because death occurs soon after consultation, end-of-life preferences are often not met, such as dying in the preferred place of death. In addition, patients’ end-of-life preferences are mainly focused on the last days and weeks before death and do not focus on future care, which ACP aims to discuss to ensure that end-of-life care meets the patients’ preferences. Since most consultations are requested to perform ACP, healthcare professionals could benefit from education in timely identifying palliative care needs, creating a shared vision on when ACP should be initiated and training in initiating palliative care. This could help general proffesionals to initiate ACP discussions themselves, as specialist palliative care teams do not have the capacity to cover all ACP discussions. In addition, patient-related factors that might identify a need for palliative care team consultation (for instance an acute hospital admission because of foreseeable symptom burden for patients with life threatening illnesses) need to be defined to ensure a more prevention-oriented approach to palliative care that allows crisis situations to be discussed in advance so that patients’ end-of-life preferences can be met.

## Supporting information

S1 Protocol (PDF)

## Abbreviations

ACP: Advance care planning
ER: Emergency room
MDT: Multidisciplinary team meeting
SD: Standard deviation
