# Supplementary material for: Current practice of hospital-based palliative care teams: Advance care planning in advanced stages of disease: A retrospective observational study
Source: PLoS One. 2024 Feb 29;19(2):e0288514. doi: 10.1371/journal.pone.0288514 (PMC10903912; doi:10.1371/journal.pone.0288514)
Supplement: S1 Protocol — (PDF) [file pone.0288514.s001.pdf]

Opportunities and threats of a hospital-based transitional palliative care team

Opportunities and threats of a hospital-based transitional palliative care team

**Protocol ID**	**Opportunities and threats of a hospital-based transitional palliative care team**
**Short title**	**Transitional palliative care team**
**EudraCT number**	***NA***
**Version**	**2.0**
**Date**	**29-07-2016**
**Principal investigator**	***Dr. B.M. Buurman***<br>AMC, inwendige geneeskunde, afd geriatrie kamer F4-159-1, Meibergdreef 9, 1105 AZ Amsterdam<br>b.m.vanes@amc.nl / 020-5565882
**Coordinating investigators**	**Dr. M. Poels**<br>AMC, inwendige geneeskunde, afd geriatrie kamer F4-137, Meibergdreef 9, 1105 AZ Amsterdam<br>m.poels@amc.nl / 020-5665984<br>**Drs. M.A. (Marike) de Meij, huisarts**<br>OLVG palliatief team<br>AMC Expertisecentrum Palliatieve Zorg<br>m.a.demeij@olvg.nl
**Subsidising party**	***ZonMW***
**Laboratory sites**	***NA***
**Pharmacy**	***NA***

TABLE OF CONTENTS

1. INTRODUCTION AND RATIONALE 6
2. OBJECTIVES 6
3. STUDY DESIGN 7
..... 7
..... 7
4. STUDY POPULATION 7
   4.1 Population (base) 7
   4.2 Inclusion criteria 7
   4.3 Exclusion criteria 7
   4.4 Sample size calculation 8
5. TREATMENT OF SUBJECTS 8
6. INVESTIGATIONAL PRODUCT 8
7. NON-INVESTIGATIONAL PRODUCT 8
8. METHODS 8
   8.1 Study parameters/endpoints 8
   Endpoints 8
   8.1.1 Other study parameters (if applicable) 9
   8.2 Randomisation, blinding and treatment allocation 9
   8.3 Study procedures 9
   8.4 Withdrawal of individual subjects 10
9. SAFETY REPORTING 10
10. STATISTICAL ANALYSIS 10
11. ETHICAL CONSIDERATIONS 10
   11.1 Regulation statement 10
   11.2 Recruitment and consent 10
   11.3 Objection by minors or incapacitated subjects (if applicable) 11
   11.4 Benefits and risks assessment, group relatedness 11
   11.5 Compensation for injury 11
   11.6 Incentives (if applicable) 11
12. ADMINISTRATIVE ASPECTS, MONITORING AND PUBLICATION 11
   12.1 Handling and storage of data and documents 11
   12.2 Monitoring and Quality Assurance 11
   12.3 Amendments 11
   12.4 Annual progress report 11
   12.5 Temporary halt and (prematurely) end of study report 12
13. STRUCTURED RISK ANALYSIS 12
14. REFERENCES 12

LIST OF ABBREVIATIONS AND RELEVANT DEFINITIONS

**ABR**	**ABR form, General Assessment and Registration form, is the application form that is required for submission to the accredited Ethics Committee (In Dutch, ABR = Algemene Beoordeling en Registratie)**
**AE**	**Adverse Event**
**AR**	**Adverse Reaction**
**CA**	**Competent Authority**
**CCMO**	**Central Committee on Research Involving Human Subjects; in Dutch: Centrale Commissie Mensgebonden Onderzoek**
**CV**	**Curriculum Vitae**
**DSMB**	**Data Safety Monitoring Board**
**EU**	**European Union**
**EudraCT**	**European drug regulatory affairs Clinical Trials**
**GCP**	**Good Clinical Practice**
**IB**	**Investigator's Brochure**
**IC**	**Informed Consent**
**IMP**	**Investigational Medicinal Product**
**IMPD**	**Investigational Medicinal Product Dossier**
**METC**	**Medical research ethics committee (MREC); in Dutch: medisch ethische toetsing commissie (METC)**
**(S)AE**	**(Serious) Adverse Event**
**SPC**	**Summary of Product Characteristics (in Dutch: officiële productinfomatie IB1-tekst)**
**Sponsor**	**The sponsor is the party that commissions the organisation or performance of the research, for example a pharmaceutical company, academic hospital, scientific organisation or investigator. A party that provides funding for a study but does not commission it is not regarded as the sponsor, but referred to as a subsidising party.**
**SUSAR**	**Suspected Unexpected Serious Adverse Reaction**
**Wbp**	**Personal Data Protection Act (in Dutch: Wet Bescherming Persoonsgevens)**
**WMO**	**Medical Research Involving Human Subjects Act (in Dutch: Wet Medisch-wetenschappelijk Onderzoek met Mensen**

SUMMARY

Rationale: The quality of hospital-based care for patients with palliative care needs is still suboptimal. Identification of palliative care needs and timely discussion of the patients' wishes and preferences often not take place. It was stated by the SONCOS working group, that from 2017 on, hospitals need to have a palliative care team available. Studies suggest that when a GP is coordinator of palliative care of a patient, unwanted hospitalizations may be prevented. Transitional palliative care teams (based in the hospital, with a GP and/or home care services in the team) might therefore be the solution. The OLVG has such a transitional team and the current study aims to explore the work of this team

Objectives:

- To explore reasons of hospitalizations of patients for whom a palliative team was consulted in the hospital;
- To explore why, when and by whom the palliative team was consulted;
- To explore needs and expectations of the patient with regard to the palliative care team;
- To investigate whether agreements with and wishes of patients (with regard to hospitalizations, treatments and preferred place of death) are fulfilled after they had a consultation from the palliative care team;
- To investigate prognoses estimated by nurse specialist with actual time to death;
- To explore communication between the palliative care team and GPs and home care/care homes;
- To explore which % of consultations is financed and what are reasons for not financing a consult (according to DBC- registrations)

Study design: Retrospective study

Study population: adults, treated in the OLVG hospital, consultation by the palliative care team.

Main study parameters/endpoints:

Reason for hospitalization

Nature and extent of the burden and risks associated with participation, benefit and group relatedness: There is no burden for the patient as this is a retrospective study design.

1. INTRODUCTION AND RATIONALE

The quality of hospital-based care for patients with palliative care needs is still suboptimal.
Identification of patients with palliative care needs and end-of-life-preference discussions often do not take place (1, 2). Furthermore, there is no clear plan for the treatment of symptoms when the patient is at home. This may result in undesirable unplanned hospital readmissions in the last phase of life (3). Also, certain procedures (such as ascites paracentesis) may well be performed in primary care by a trained doctor, however when trained doctors are not available, the patient will be admitted to the hospital for this procedure.
Currently, an increasing number of hospitals form a palliative care team. The Dutch foundation for oncology collaboration (SONCOS) even added this as a requirement for cancer centres, becoming effective from January 1st, 2017 on. With these norms, hospitals are required to have a multidisciplinary palliative team that measures patients' palliative care need and is accessible for consultation. The goals of these palliative teams are twofold: to improve quality of intramural palliative care and to improve communication and collaboration with primary care (4).
There is evidence that the number of people who die in the hospital and the number of transfers in the last three months of life are lower when a GP is the main coordinator of (palliative) care and if there are sufficient resources in primary care (5). Therefore, expanding the hospital-based team to a transitional palliative care team that consists not only of hospital professionals, but also professionals working in primary care (GP and community care nurse, both specialized in palliative care) might help tackle the described issues.
The OLVG hospital in Amsterdam currently has a transitional palliative care team and the number of consultations is rapidly increasing over the years. Approximately 75% of the consults takes place during hospitalization and the rest in the outpatient department. Their structured approach to the wishes and preferences of the patient as their primary priority, and good communication with the GP is an ideal example for hospital based transitional palliative care teams. The OLVG has such a transitional team and the current study aims to explore the work of this team.

2. OBJECTIVES

In this exploratory study, we will investigate opportunities and threats of a (transitional) palliative care team. Objectives:

- To explore reasons of hospitalizations of patients for whom a palliative team was consulted in the hospital;
- To explore why, when and by whom the palliative team was consulted;
- To explore needs and expectations of the patient with regard to the palliative care team;
- To investigate whether agreements with and wishes of patients (with regard to hospitalizations, treatments and preferred place of death) are fulfilled after they had a consultation from the palliative care team;
- To investigate prognoses estimated by nurse specialist with actual time to death;

- To explore communication between the palliative care team and GPs and home care/care homes;
- To explore which % of consultations is financed and what are reasons for not financing a consult (according to DBC- registrations)

3. STUDY DESIGN

Retrospective analyses of patients with a palliative care team consultation between January and May 2016 in the OLVG location East.

We will analyze data on the period of the consultation itself, 6 months prior to first consultation (prior hospitalizations and disease history) and 6 months after first consultation (follow up).

Timeline (recruitment of new consults of the palliative care team between January and May 2016)

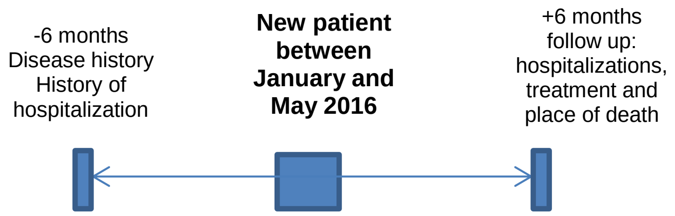

4. STUDY POPULATION

4.1 Population (base)

Patients of whom the palliative care team was consulted for the first time between January and May 2016 with a complete consultation of the palliative care team

4.2 Inclusion criteria

In order to be eligible to participate in this study, a subject must meet all of the following criteria:

- Adult (≥18 years)
- Consulted by the palliative care team

4.3 Exclusion criteria

A potential subject who meets any of the following criteria will be excluded from participation in this study:

- Incomplete consult without discussion in multidisciplinary meeting

4.4 Sample size calculation

Due to the exploratory nature of this study, a sample size calculation is not possible. In our opinion, a sample of 50-60 patients will be sufficient to explore the objectives of this study. With 175 complete consultations in 2015, inclusion of patients in the first 4 months of 2016 will result in a sample between 50-60 patients.

5. TREATMENT OF SUBJECTS

NA

6. INVESTIGATIONAL PRODUCT

NA

7. NON-INVESTIGATIONAL PRODUCT

NA

8. METHODS

Objectives:

- To explore reasons of hospitalizations of patients for whom a palliative team was consulted in the hospital;
- To explore why, when and by whom the palliative team was consulted;
- To explore needs and expectations of the patient with regard to the palliative care team;
- To investigate whether agreements with and wishes of patients (with regard to hospitalizations, treatments and preferred place of death) are fulfilled after they had a consultation from the palliative care team;
- To investigate prognoses estimated by nurse specialist with actual time to death;
- To explore communication between the palliative care team and GPs and home care/care homes;
- To explore which % of consultations is financed and what are reasons for not financing a consult (according to DBC- registrations)

8.1 Study parameters/endpoints

Endpoints

- Reasons for hospitalization of patients for whom a palliative team was consulted during hospitalization. With use of an expert panel, we will discuss preventability of these hospitalizations in the analysis phase of the study.
- Timing& reasons for palliative team consultation & profession of person who consulted the palliative care team
- Needs and expectations of patients with regard to palliative care team consultation

- Fulfilment of agreements with and wishes of patients (with regard to hospitalizations, treatments and preferred place of death)
    - Agreements available/wishes noted during consults with palliative care team regarding future hospitalizations, treatments and preferred place of death
    - Agreements and wishes fulfilled 6 months after consultation?
- Estimated prognose by nurse specialist and actual time to death
- communication of the palliative care team with GPs and home care/carehomes:
    - Timing & amount of communication
    - Oral/written communication
    - Content of handover
- Financing of consultations and reasons for not financing a consult (according to DBC-registrations)

8.1.1 Other study parameters (if applicable)

- Date of birth
- Diagnosis + timing of diagnosis
- Number of acute hospitalizations in 6 months before consultation of palliative care team
- If inpatient clinic: hospitalization department
- Number of consults by palliative care team
- Number and type of specialists during MDO meeting where patient is discussed

8.2 Randomisation, blinding and treatment allocation

NA

8.3 Study procedures

As this is a retrospective data analyses study, informed consent will not be required.
The palliative care team hands over a list of new consults in the period between January and May 2016. The researcher appoints a research number to each subject on the list. With use of the electronic medical file and insight into DBC registration, the following variables will be collected:

In 6 months before inclusion	**Consultation: characteristics**	6 months follow up
Acute hospitalizations<br>Disease/treatment history	- Date of birth<br>- Diagnosis<br>- Date of diagnosis<br>- Date of PT consultation<br>- Reason for PT consultation<br>- Number of consults by PT during	- Hospitalizations<br>- Emergency department visits<br>- Treatment (when wishes with regard to treatment were noted)

	hospitalization<br>- Wishes and expectations of the patient with regard to the palliative care team<br>- Agreements/wishes of patients noted with regard to hospitalization/ treatment / preferred place of dying<br>- Prognosis (estimated by nurse specialist)<br>- Contact between PT and GP/home care/care home: number of contacts, method, handover<br>For inpatient clinic:<br>- Hospitalization department<br>- Reason for hospitalization	- Date and place of death<br>- DBC financing: DBC registered & approved?

8.4 Withdrawal of individual subjects

NA

9. SAFETY REPORTING

NA

10. STATISTICAL ANALYSIS

Descriptive statistics (mean±SD, median (IQR) or count (%)) will be used to describe the study sample with regard to all outcomes.

11. ETHICAL CONSIDERATIONS

11.1 Regulation statement

The study will be conducted according to the principles of the Declaration of Helsinki, the 7th revision (2013) and in accordance with the Medical Research Involving Human Subjects Act (WMO) and other guidelines, regulations and Acts

11.2 Recruitment and consent

The nurses of the palliative care team of the OLVG will hand over a list of patients consulted by the palliative care team between January and May 2016. Because of the retrospective design and poor prognosis of the patients, informed consent is not necessary and / or possible.

11.3 Objection by minors or incapacitated subjects (if applicable)

NA

11.4 Benefits and risks assessment, group relatedness

This study will give insight into the work of the palliative care team. A report with results will be send to the palliative care team and hospital directors in order to improve the work of the team.

11.5 Compensation for injury

NA

11.6 Incentives (if applicable)

NA

12. ADMINISTRATIVE ASPECTS, MONITORING AND PUBLICATION

12.1 Handling and storage of data and documents

Data will be handled confidentially and anonymously. Each recruited patient will get a research number. A secured subject identification code list will be used which is only accessible by the principle investigator.

12.2 Monitoring and Quality Assurance

NA

12.3 Amendments

Substantial amendments which would change the nature of the study will be notified to the METC that gave a favourable opinion.

Non-substantial amendments will not be notified to the accredited METC and the competent authority, but will be recorded and filed by the sponsor.

12.4 Annual progress report

The sponsor/investigator will submit a summary of the progress of the trial to the accredited METC once a year. Information will be provided on the date of inclusion of the first subject, numbers of subjects included and numbers of subjects that have completed the trial, serious adverse events/ serious adverse reactions, other problems, and amendments.

12.5 Temporary halt and (prematurely) end of study report

Within one year after the end of the study, the investigator/sponsor will submit a final study report with the results of the study, including any publications/abstracts of the study, to the accredited METC.

13. STRUCTURED RISK ANALYSIS

NA

14. REFERENCES

1. Buurman BM, van Munster BC, Korevaar JC, Abu-Hanna A, Levi M, de Rooij SE. Prognostication in acutely admitted older patients by nurses and physicians. Journal of general internal medicine. 2008;23(11):1883-9.
2. Claessen SJ, Francke AL, Echteld MA, Schweitzer BP, Donker GA, Deliens L. GPs' recognition of death in the foreseeable future and diagnosis of a fatal condition: a national survey. BMC family practice. 2013;14:104.
3. De Korte-Verhoef MC, Pasman HR, Schweitzer BP, Francke AL, Onwuteaka-Philipsen BD, Deliens L. Reasons for hospitalization at the end of life: differences between cancer and non-cancer patients. Supportive care in cancer : official journal of the Multinational Association of Supportive Care in Cancer. 2014;22(3):645-52.
4. (SONCOS) SOS. 4e normeringsrapport. 2016.
5. Pivodic L, Pardon K, Morin L, Addington-Hall J, Miccinesi G, Cardenas-Turanzas M, et al. Place of death in the population dying from diseases indicative of palliative care need: a cross-national population-level study in 14 countries. Journal of epidemiology and community health. 2016;70(1):17-24.
